# Cannabidiolic acid in Hemp Seed Oil Table Spoon and Beyond

**DOI:** 10.3390/molecules27082566

**Published:** 2022-04-15

**Authors:** Ersilia Nigro, Maria Tommasina Pecoraro, Marialuisa Formato, Simona Piccolella, Sara Ragucci, Marta Mallardo, Rosita Russo, Antimo Di Maro, Aurora Daniele, Severina Pacifico

**Affiliations:** 1 Dipartimento di Scienze e Tecnologie Ambientali, Biologiche, Farmaceutiche, Università della Campania “Luigi Vanvitelli”, Via Vivaldi 43, 81100 Caserta, Italy or nigro@ceinge.unina.it (E.N.); mariatommasina.pecoraro@unicampania.it (M.T.P.); marialuisa.formato@unicampania.it (M.F.); sara.ragucci@unicampania.it (S.R.); simona.piccolella@unicampania.it (S.P.); marta.mallardo@unicampania.it (M.M.); rosita.russo@unicampania.it (R.R.); antimo.dimaro@unicampania.it (A.D.M.); 2 CEINGE, Biotecnologie Avanzate Scarl, Via Gaetano Salvatore 486, 80145 Naples, Italy; 3 Dipartimento di Medicina Molecolare e Biotecnologie Mediche, “Federico II” Università degli Studi di Napoli, 80131 Naples, Italy

**Keywords:** Cannabidiolic acid, industrial hemp, keratinocytes, cytotoxicity, cytokines, antimicrobial activity

## Abstract

Cannabidiolic acid (CBDA) is the main precannabinoid in industrial hemp. It represents a common constituent of hemp seed oil, but mainly abundant in the aerial parts of the plant (including their processing waste). Thus, the optimization of fast and low-cost purification strategies is mandatory, as well as a deep investigation on its nutraceutical and cosmeceutical properties. To this purpose, CBDA content in hemp seed oil is evaluated, and its recovery from wasted leaves is favorably achieved. The cytotoxicity screening towards HaCaT cells, by means of MTT, SRB and LDH release assays, suggested it was not able to decrease cell viability or perturb cell integrity up to 10 μM concentration. Thus, the ability of CBDA to differentially modulate the release of proinflammatory cytokines and chemokines mediators has been evaluated, finding that CBDA decreased IFN-γ, CXCL8, CXCL10, CCL2, CCL4 and CCL5, mostly in a dose-dependent manner, with 10 μM tested concentration exerting the highest activity. These data, together with those from assessing antimicrobial activity against Gram(+) and Gram(−) bacteria and the antibiofilm formation, suggest that CBDA is able to counteract the inflammatory response, also preventing bacteria colonization.

## 1. Introduction

Nutrition greatly benefits our health, and the diet, when well-balanced and properly enriched in bioactive substances, is capable of providing, beyond energy and nutrients, phytobioactive agents, useful to meet the needs of the body as a whole and of individual tissues specifically [1]. The food nutritional and nutraceutical value could nourish the skin [2], which is characterized by a high turnover, also from the inside, favoring a new form of nutrition or rather nutritional supplementation, known as nutricosmetic, which aims to optimize the needs of the skin satisfying it [3]. Thus, through a joint approach between dermo-cosmetic and food research, food supplements are prompt to promote skin hydration, also improving skin tissues fluidity, and increasing skin tone brightness and uniformity, and delaying skin aging [4,5,6]. In this context, food rich in essential fatty acids (EFA) could be a useful player in epidermal integrity maintenance, so much so that and it was found that the topical application of plant oils is a strategy to protect skin, retaining its moisture [7], and α- and/or γ-linolenic-enriched foods are suggested to counteract epidermal hyper-proliferation, and thus dry skin conditions and mild atopic dermatitis [8,9]. In this framework, hemp seed oil is actually of great interest [10], together with its richness in n-3 and n-6 essential fatty acids, mainly linoleic acid and α-linolenic acid. Indeed, beyond essential polyunsaturated fatty acids, hemp seed oil contains important minor components such as phytosterols, the various vitamin E vitamers (e.g., α-, δ- and γ -tocopherols), phenolamides and other antioxidant polyphenols [11,12]. All these substances in a single spoon could be very useful aids for the skin, and in the actual hemp revival, the hemp seed oil is ready to find new perspectives of use, beyond those expected in the food and nutraceutical sector [13]. To this purpose, a special attention should be paid to cannabinoids, mainly to Cannabidiolic acid (CBDA), which is the most abundant precannabinoid compound in industrial hemp [14], that is all the *Cannabis sativa* varieties, cultivated for food purposes [15], for the resistant fibers and/or for the medical virtues [16,17]. The anti-inflammatory activity of cannabinoids is the object of interesting research that point out some of these compounds as efficacious in slowing down different skin disorders [18]. However, the literature is full of contradictory data, and promising findings are related to cannabinoids as good agents against atopic dermatitis, psoriasis, acne vulgaris, pruritus, etc. [19]. Cannabidiol appears as the main actor [20], mainly through its antioxidant capability, and the downregulation of pro-inflammatory chemokines/cytokines [21].

Precannabinoids are an integral part of the chemical composition of the hemp fruit, commonly known as hemp seed or “canapuccia”. This edible achene contains in its external shell bioactives, such as precannabinoids and phenolamides, and an internal part consisting of the precious aleurone bodies due to the high content of polyunsaturated fatty acids of the n:6 and n:3 series and proteins [22,23]. Thus, the oil from cold-pressing or other extractive methods could contain acid phytocannabinoids, whose amount in the edible product is strongly affected by the achene ripeness level. In fact, unripe fruits increase their content in precannabinoids, whereas these compounds are not detectable in oils from dehulled seeds [24]. The production of the edible hemp seed oil and its by-products whose transformation into flour is suitable for the preparation of nutritious baked goods [17], could not ignore Cannabidiolic acid, which should be addressed in a new nutraceutical and nutricosmetic perception. Indeed, it appears clear that until now CBDA chemistry and above all its bioactivity failed to arouse interest, when compared to those of the more stable neutral derivative, cannabidiol (CBD), which is subject of numerous pharmacological investigations that have dictated its use for the treatment (not only symptomatic) of several morbid conditions [25]. In this context, the identification of this molecule in the hemp seed, starting from its oil, is reported, in conditions that go beyond the possible seed contamination with aerial parts-deriving residues. However, the possibility of isolating Cannabidiolic acid from hemp different organs and products with high yield cannot be neglected also to enhance its nutraceutical and/or cosmeceutical use. The reliable bioactivity of CBDA was suggested by analyzing its cytotoxicity, and the ability to differently module cytokines and chemokines in the immortalized HaCaT keratinocytes. The antimicrobial activity against Gram(+) and Gram(−) bacteria is also reported.

## 2. Results and Discussion

### 2.1. CBDA, a Nutraceutical Value in the Hemp Seed Oil Table Spoon and Hemp Wastes

**Figure 1:**
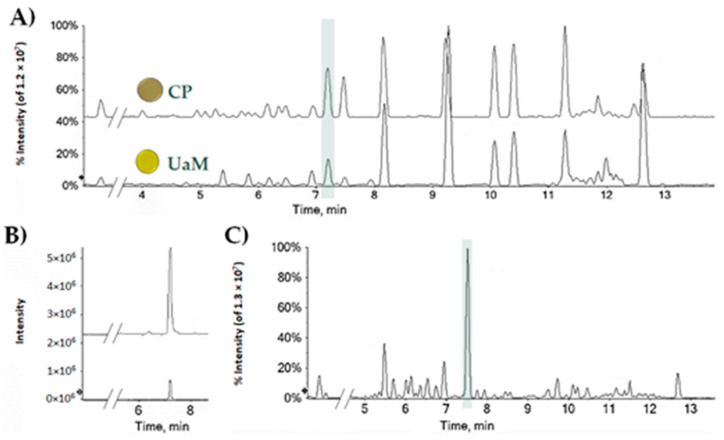
(**A**) TICs (Total Ion Chromatograms) of UaM and CP hemp seed oils; (**B**) XICs (eXtracted Ion Chromatograms) of the ion at *m/z* 357.2071 ± 0.0025; (**C**) representative TIC of leaf hemp sample collected at seed harvesting time.

Hemp seed oil contains Cannabidiolic acid, whose amount strongly depends on the extractive method applied, hemp variety, pedoclimatic condition in the collection site, etc. [26]. Herein, the CBDA quantitation in oils from the industrial hemp Futura 75 variety, was carried out. To this purpose, oils were obtained through cold-pressing and ultrasound-assisted maceration, and profiled by means of UHPLC-QqTOF-ESI-MS/MS techniques. Both the oils are characterized by the typical pattern of polyunsaturated fatty acids, among which linoleic acid and α-linolenic acid are the most abundant, whereas CBDA was the main constituent among the minor ones (Figure 1A,B and Figure S1). Taking into account the calibration curve of CBDA, prepared using different concentrations of the pure compound, as it was previously isolated and deeply characterized from inflorescences in our lab [27], the amount of CBDA was found to be equal to 0.18 ± 0.01 mg per g of cold-pressing oil, and 0.03 ± 0.001 mg per g of UaM-extracted oil, which corresponded to 7.5 ± 0.25 µg per g of seed, taking into account the UaM extraction yield. Indeed, after seed harvesting, hemp leaves, constituting the residual biomass, should be disposed or utilized to serve different sectors. Food and nutraceutical sectors could benefit of the recovery of this hemp waste to achieve CBDA and other bioactive compounds, usable as antioxidant and/or antimicrobial additives. In particular, the ultrasound-assisted maceration in *n*-hexane of leaves provided a leaf extract, whose profile was in line with a highly feasible CBDA recovery (Figure 1C and Figure S1), which was exploitable along the entire cultivation period. At hemp seed harvesting time, collecting also leaf samples, and extracting them, it was found a content equal to 0.81 ± 0.03 mg per g of dried leaf. This maximized the feasibility of CBDA recovery, leading to its purification according to Formato et al. [27] and briefly summarized below (see Section 3.1). The purity of isolated CBDA was verified by reversed phase liquid chromatography coupled to mass spectrometry and UV spectrophotometric detection. In the Supplementary Materials, UHPLC-TOF-MS and HPLC-UV chromatograms, and also a UV-Vis spectrum of isolated CBDA, are provided (Figures S2 and S3).

### 2.2. CBDA Cytotoxicity in HaCaT Cells

**Figure 2:**
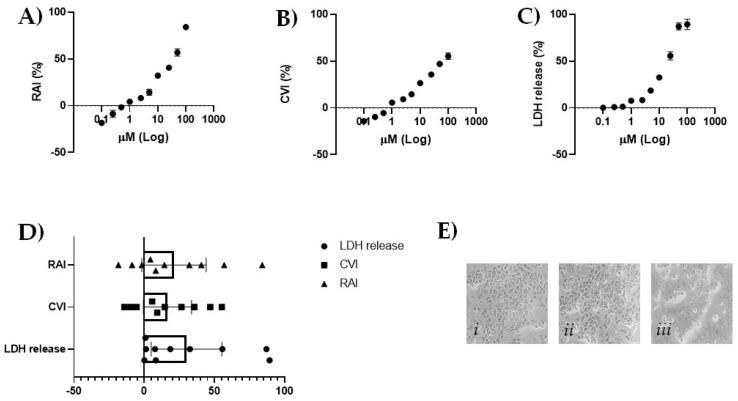
(**A**) Redox mitochondrial activity inhibition (%) by means of MTT data; (**B**) cell viability inhibition (%) by SRB assay data; (**C**) LDH release (%) by LDH assay data. Values are the mean ± SD of two independent experiments. Six replicates were performed in each experiment. (**D**) Scatter plot of data from the three different cytotoxicity assays with a representation of their 95% confidence limits using Graphpad Prism 8 software (Graphpad Software, La Jolla, CA, USA). (**E**) Representative images, acquired by Inverted Phase Contrast Brightfield Zeiss Primo Vert Microscope, of (**i**) untreated HaCaT cells; (**ii**) CBDA 2.5 μM treated HaCaT cells; (**iii**) CBDA 25 μM treated HaCaT cells.

In order to hypothesize the nutraceutical use of CBDA for skin, the cytotoxic effect of CBDA was preliminarily evaluated by means of three different assays towards the HaCaT cell line, which derives from normal human skin and it is spontaneously immortalized [28]. Evaluating CBDA cytotoxicity in HaCaT cells at ten different concentrations (from 0.1 to 100 μM), it was observed that it was able to exert a dose-dependent inhibition of mitochondrial redox activity. The compound appeared to be non-toxic when tested at low concentrations (up to 10 μM), whereas it was able to reduce dehydrogenase activity by 40.7%, 57%, and 84% at 25, 50, and 100 μM (Figure 2A). This was not in line with Martinenghi et al. [29], who observed that CBD showed dose-dependent killing up until 16 µg/mL, where at higher doses the cytotoxicity was stabilized at 50%. The increase in concentration appeared to affect cell morphology, pointing that in dosages above 10 μM, cells were similar to balloon-like bubbles [30].

When sulforhodamine B assay was performed, CBDA at 10 and 25 μM was observed to decrease by 26.5% and 35.7%, respectively, the total protein mass, which is related to cell number, whereas at the lowest tested dose there was no inhibition of cell viability (Figure 2B). However, the inhibitory effect does not increase in proportion to the dose increase, with an inhibition of 55.3% occurring in cells treated with CBDA at 100 μM. At this latter concentration, as well as at 50 μM, a marked lactate dehydrogenase (LDH) release was further detected (Figure 2C). The intracellular LDH enzyme was from damaged cells and its content is an indicator of cell membrane integrity. The effect observed in the highest concentration of CBDA-tested HaCaT cells was unrelated to what was recently found testing an extract of Cannabis sativa L. herb [31]. The neutral derivative of CBDA was previously tested on keratinocytes and, according to our data, no cytotoxic effects were at 1, 5, 10, and 20 μM after 6, 12, and 24 h [32]. Furthermore, it was reported that CBD was able to induce keratinocyte differentiation, and skin development [33]. The extensive investigation of the CBD efficacy in maintaining skin health [34] does not find a counterpart in its precannabinoid, which has attracted less attention, probably due to its tendency to decarboxylate or, more simply, to the lack of awareness of its presence and relative abundance. Thus, only indirect data are available on CBDA activity in skin cells. Recently, an hemp seed apolar extract, with CBD and CBDA as the main compounds, was reported to exert anti-inflammatory and anti-lipogenic activity in a Propionibacterium acnes-triggered inflammation and lipogenesis model [35]. Considering that both CBDA and CBD have structurally low binding affinity to the endocannabinoid receptors, the less polarity of CBDA was not limiting for microemulsion formulations for transdermal delivery [36].

### 2.3. CBDA Modulated Cytokines and Chemokines in HaCaT Cells

**Figure 3:**
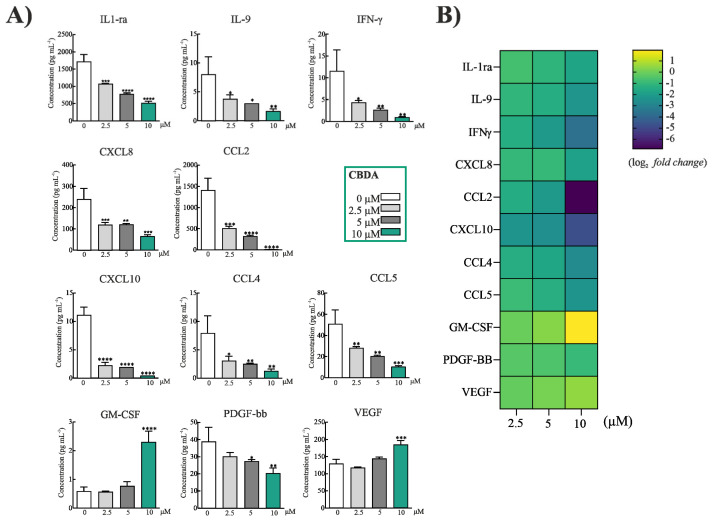
(**A**) Cytokines and chemokines profile in HaCaT cells treated with CBDA 2.5, 5 and 10 μM, and in untreated cells serving as control. Statistical analysis was performed using Graphpad Prism 8 software (Graphpad Software, La Jolla, CA, USA). The results were statistically analyzed using one-way ANOVA test followed by Dunnett’s Multiple Comparison Test. (*) *p* < 0.05; (**) *p* ≤ 0.01; (***) *p* ≤ 0.001 and (****) *p* ≤ 0.0001 indicate a statistically significant difference with controls. (**B**) Heatmap showing the log_2_ fold change of differentially expressed cytokines in culture media of HaCaT cells after treatment with CBDA. Color codes in each panel refer to blue for lower expression (down-regulation) and yellow for higher expression (up-regulation) levels.

Since the HaCaT cell line is a reliable model to screen for new anti-inflammatory compounds for skin diseases, and keratinocytes are involved in the initiation and perpetuation of the cutaneous inflammatory response [37], the study of the ability of CBDA to differentially modulate the release of proinflammatory mediators was carried out (Figure 3A). For this purpose, HaCat cells were first treated with the non-toxic doses 2.5, 5, and 10 μM. Cytokines, including chemokines, play a critical role in mediating inflammation, and they are involved in cell communication, also regulating immunity, and cell activation, migration, and proliferation [38]. The CBDA concentration appeared to be determinant in the response observed. In fact, it was observed that CBDA inhibited dose-dependently IL-1ra (IL-1 receptor antagonist), a regulator of IL-1 signaling, and a competitive antagonist of the IL-1 agonist receptor (IL-1R1) [39]. The inhibition of the pleiotropic cytokine interleukin-9 (IL-9) also occurred. T cells and mast cell growth factor are implicated in allergic and autoimmune disorders, parasitic infections, and antitumor immunity [40]. Recently, it was observed that an increased IL-9 secretion is related to atopic dermatitis anthogenesis [41]. Moreover, CBDA was able to decrease IFN-γ, CXCL8, CXCL10, CCL2, CCL4 and CCL5. With the only exception of the effect on CXCL8, towards which a similar response was from CBDA 2.5 and 5 μM treatments, all the other mediators were extensively inhibited in a dose-dependent manner, with CBDA at 10 μM exerting the highest activity as also evidenced by the heatmap (Figure 3B). CBDA appeared to effectively exert the downregulation of IFN-γ, which is the dominant cytokine in inflamed skin, and in the skin of patients with atopic dermatitis, where it mediates also the upregulation of CCL5 expression [42]. The latter is a proinflammatory chemokine, formerly also known as RANTES, whose expression increases in lesioned skin [43], especially in lesions of psoriasis [44]. CCL5 expression detected in the supernatant of HaCaT cells was reduced by 20%, following CBDA 10 μM treatment. The role of IL-8 in psoriatic skin, as well as in atopic dermatitis, also was elucidated [45], and effect of CBDA at 10 μM, which was able to reduce IL-8 by 32%, is non-negligible. IL-8 is involved and produced in many inflammatory reactions, and keratinocytes are a rich source of it [46]. While CCL4, also known as macrophage inflammatory protein-1β (MIP-1β), was decreased by CBDA treatment in the HaCaT cells, CCL2 (MCP-1, Monocyte chemoattractant peptide-1), a chemokine acting as mediator in allergic rhinitis and psoriasis [47], and able to mediate inflammation after tissue damage in various organs, was completely pulled down, with a three-fold decrease compared to untreated cells already following the treatment with CBDA 2.5 μM. Finally, a marked decrease was for CXCL10, claimed as marker of vitiligo and psoriasis [48,49]. Contrary to what previously demonstrated for CBD, an increase in GM-CSF was found, which was poorly contained in untreated cells, as well as in CBDA 2.5 and 5.0 μM treated cells. Vascular endothelial growth factor (VEGF), which was reported to modulate HaCaT cells adhesion [50], appeared to be stimulated by the highest CBDA concentration, which further slowed down the platelet-derived growth factor (PDGF), commonly involved in epithelial hyperproliferation [51]. Recent findings showed a similar cytokine and chemokine behavior in CBD-treated blood of depressed patients, concluding that cannabidiol exerts dose-dependent immunomodulatory effects, and that at higher concentrations it was able to worsen inflammation [52].

### 2.4. Antibacterial Activity of CBDA: MIC and MBC Determination

Disturbance in the inflammatory and immune systems mediated by the above-considered cytokines and chemokines, leads very often to episodes of opportunistic skin bacterial infections compromising skin health [53]. There is the urgent need to discover compounds acting on both the inflammatory response and the prevention of bacteria colonization. Therefore, the antibacterial activity of CBDA, also considering recent literature data [29,54] was tested ranging from 0.064 to 64 µg/mL against two Gram-negative bacteria such as *P. aeruginosa* ATCC 27853 and *E. coli* ATCC 13762 and two Gram-positive bacteria such as *S. aureus* ATCC 6538 and *E. faecalis* ATCC 29212. The MIC values of CBDA ranged from 0.5 to 4 µg/mL for all the microorganisms tested, with the exception of *P. aeruginosa* that is resistant up to a concentration of 8 µg/mL (Table 1). CBDA also showed bactericidal effects against both Gram-negative and Gram-positive bacteria used for the study as no growth of bacteria was observed at the different concentrations reported in Table 1 (MBC values).

### 2.5. Evaluation of Antimicrobial Activity of CBDA by Time-Killing Assay

**Figure 4:**
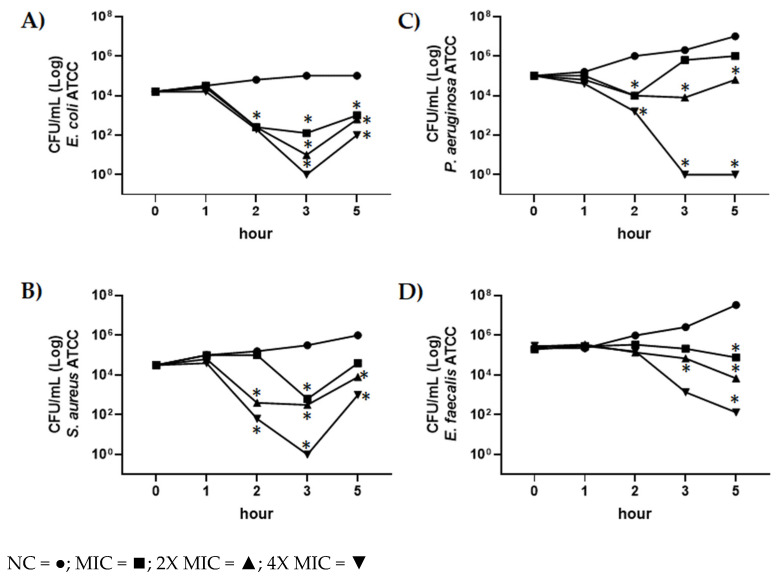
Time-kill kinetics of CBDA against *E. coli* ATCC 13762, *S. aureus* ATCC 6538, *P. aeruginosa* ATCC 27853 and *E. faecalis* ATCC 29212. Time killing curve of CBDA at MIC (◼), 2X MIC (▲), and 4X MIC (▼) values against (**A**) *E. coli* ATCC 13762, (**B**) *S. aureus* ATCC 6538, (**C**) *P. aeruginosa* ATCC 27853, and (**D**) *E. faecalis* ATCC 29212. NC (●): negative control. Data were expressed as the mean ± SD of three individual experiments. Calculated statistical significance by Student t test at 1, 2, 3, 5 h vs. 0 h was indicated * *p* < 0.05.

The time killing studies of CBDA against *E. coli* ATCC 13762, *S. aureus* ATCC 6538 and *P. aeruginosa* ATCC 27853 and *E. faecalis* ATCC 29212 were performed. Different CBDA concentrations were used (i.e., MIC, 2XMIC, 4XMIC values), and bacteria were analyzed at different incubation times (1, 2, 3, 5 h); data obtained are shown in Figure 4.

During the first incubation hours (2 and 3 h), CBDA exhibited a strong reduction of the growth of *E. coli* ATCC 13762, *S. aureus* ATCC 6538, E. faecalis ATCC 29212 and *P. aeruginosa* ATCC 27853 strains at concentrations of CBDA as shown by the MIC value (Figure 4A–D). At prolonged incubation times, a slight recovery of growth for the following three bacterial strains was observed for the MIC value: *E. coli* ATCC 13762, *S. aureus* ATCC 6538, and *P. aeruginosa* ATCC 27853 (Figure 4A–C). On the contrary, for *E. faecalis* ATCC 29212, no growth recovery was observed (Figure 4D). Regarding concentrations of CBDA of 2X and 4X MIC, at prolonged incubation times a recovery of growth and vitality was observed only for *E. coli* and *S. aureus* (Figure 4A,B).

### 2.6. Biofilm Formation

**Figure 5:**
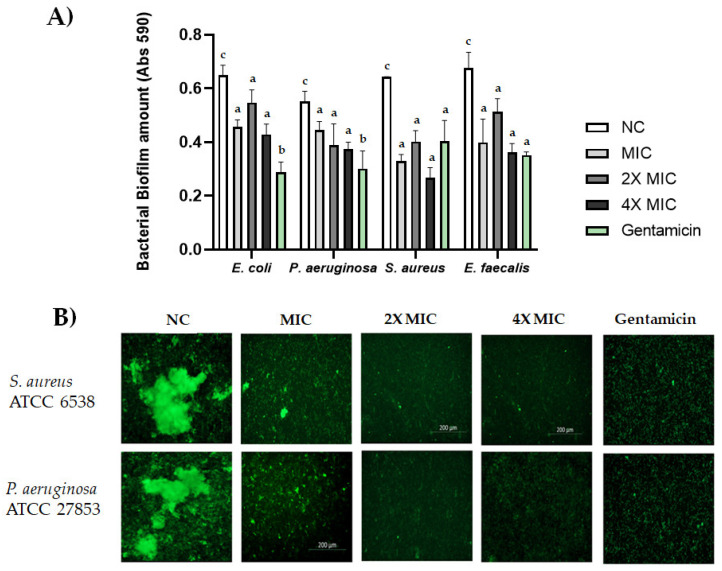
(**A**) Activity of CBDA against biofilms of *E. coli* ATCC 13762, *S. aureus* ATCC 6538, *P. aeruginosa* ATCC 27853 and *E. faecalis* ATCC 29212. CBDA was tested for inhibition of biofilm formation at concentrations corresponding to MIC, 2X MIC and 4X MIC against *E. coli* ATCC 13762, *S. aureus* ATCC 6538, *P. aeruginosa* ATCC 27853 and *E. faecalis* ATCC 29212. NC: negative control. Gentamycin (0.2 µg/mL) was used as positive control. Data were expressed as the mean ± SD of three individual experiments. Multiple comparisons were performed by ANOVA. Different letters (a, b, c) indicate significant differences at *p* < 0.05 analyzed by ANOVA. (**B**) Biofilms of *S. aureus* ATCC 6538 and *P. aeruginosa* ATCC 27853 were grown in 24-well plates for 24 h in the absence (NC) or presence of CBDA and stained with the LIVE/DEAD bacterial biofilm kit (SYTO-9 staining).

CBDA was tested for inhibition of biofilm formation using two different assays, quantified through staining with crystal violet (Figure 5A) and staining and visualization with a microscope (Figure 5B). Different concentrations (MIC, 2X MIC, 4X MIC) of CBDA were used, as shown in Figure 5A,B. CBDA was able to inhibit biofilm formation from all bacteria strains at concentrations as low as the MIC value (Figure 5A). In addition, during the biofilm formation phase, CBDA showed maximum activity at concentrations corresponding to 4X MIC for each microorganism (Figure 5A). Biofilms formed by *S. aureus* and *P. aeruginosa* after 24 h of static culture were stained with a LIVE/DEAD stain and imaged by Cell Discoverer 7, Zeiss (Figure 5B). Upon examination of the images, the biofilms formed by untreated bacteria were noticeably thicker and bigger in volume than that those formed by treated bacteria.

The inhibition of biofilm formation induced by CBDA was evident at concentrations equal to the MIC and the maximum effect was obtained at concentrations corresponding to 4X MIC for both *S. aureus* and *P. aeruginosa*. The antibiofilm formation of cannabinoids was shown for CBD, which inhibited *Candida albicans* biofilm by a multi-target mode of action [55], and in *S. aureus*, *S. pneumoniae*, and *C. difficile*, acting mainly defining membrane disruption [56].

### 2.7. Permeabilization of S. aureus ATCC 6538 and E. coli ATCC 13762 Membranes Induced by CBDA

**Figure 6:**
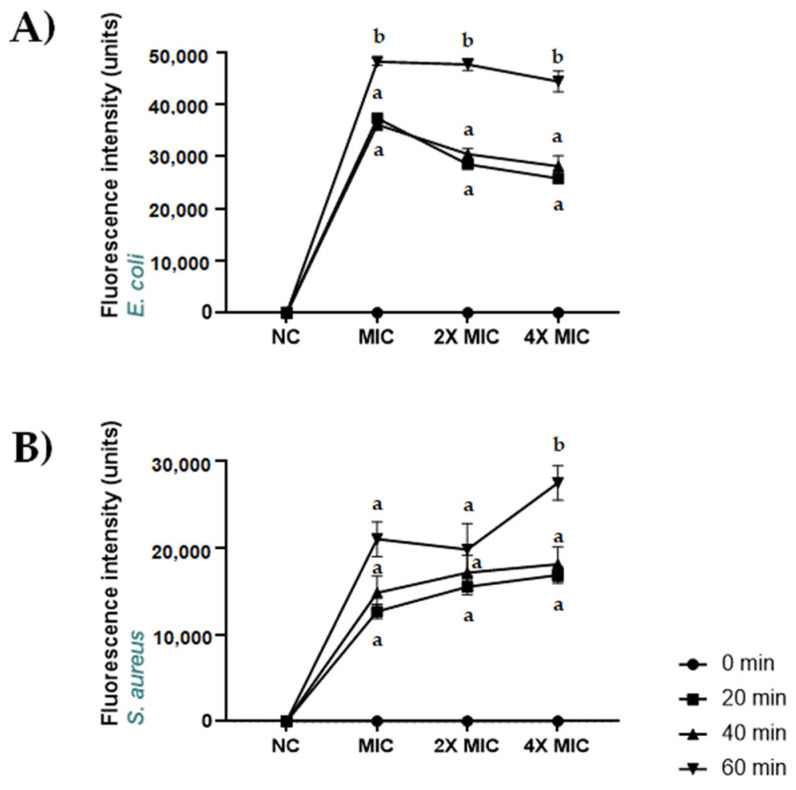
Membrane permeabilization induced by CBDA in *S. aureus* ATCC 6538 and *E. coli* ATCC 13762. Dose–response curves of membrane permeabilization of (**A**) *S. aureus* ATCC 6538 and (**B**) *E. coli* ATCC 13762, measured by SYTOX Green fluorescence. Basal values at 0 time are coincident with basal fluorescence intensity axis, where measures are indicated in a non-proportional scale. Bacterial cells were suspended in distilled water and treated with CBDA for 0, 20, 40 and 60 min, whereupon fluorescence was measured. Values are means with standard error bars of triplicate measurements and correspond to one representative experiment of the three. Multiple comparisons were performed by ANOVA. Different letters (a, b) indicate significant differences at *p* < 0.05 analyzed by ANOVA. NC: negative control.

The integrity of the plasma membranes of *S. aureus* ATCC 6538 and *E. coli* ATCC 13762 after incubation with CBDA was assessed (Figure 6). CBDA-induced permeabilization was evaluated 20, 40 and 60 min after the addition of SYTOX Green and CBDA at concentrations corresponding to MIC, 2X MIC, 4X MIC for each bacterium. CBDA caused an increase in SYTOX Green influx already after 20 min of incubation for both S. aureus and *E. coli*. The most relevant effect was obtained after 60 min of incubation with CBDA at a concentration equal to the MIC for *E. coli* and 4X MIC for *S. aureus*.

## 3. Materials and Methods

### 3.1. CBDA Quantitation in Hemp Seed Oil and Leaf Wastes

Hemp seeds (1.0 g) from Futura 75 variety, collected within ProHemPil Project activities in Campania Region (Italy), were first cryo-dried and ground, and then subjected to ultrasound assisted maceration (Branson UltrasonicsTM BransonicTM M3800-E, Danbury, CT, USA) in *n*-hexane using the ratio hemp seeds:solvent equal to 1:5. Three extraction cycles were carried out (40 min each). After solvent removal by using a rotary evaporator, the UaM hemp seed oil was obtained with an extraction yield equal to 25%. The monovarietal Futura 75 cold-pressing oil was acquired by a local producer. Hemp leaves, collected at seeds harvesting time, also were ultrasound assisted macerated. All the samples were analyzed mainly by means of UHPLC-ESI-Q*q*TOF-MS techniques. A Shimadzu NEXERA UHPLC system (Shimadzu, Tokyo, Japan) equipped with a Luna^®^ Omega Polar C18 column (1.6 μm, 50 × 2.1 mm i.d, Phenomenex, Torrance, CA, USA) was utilized and a binary solution (A) H_2_O (0.1% HCOOH), (B) CH_3_CN (0.1% HCOOH) was used for separative purposes. The gradient program initiated at 5% B, and linearly ramped up to 55% B in 5 min, to 75% B in other 5 min, and finally to 95% B in one minute. The program held for 1.0 min at 95% B, and then the initial condition was restored and held for another 2 min. The total run time was 13 min, with a flow rate of 0.4 mL min^−1^. The injection volume was 2.0 μL. MS analysis was performed using the AB SCIEX Triple TOF^®^ 4600 (AB Sciex, Concord, ON, Canada). The MS parameters were as follows: curtain gas 35 psi, nebulizer gas 60 psi, heated gas 60 psi, ion spray voltage 4.5 kV, interface heater temperature 500 °C, and declustering potential −75 V. In TOF-MS/MS experiments, collision energy applied was −55 V with a collision energy spread (CES) of 35 V. The instrument was controlled by Analyst^®^ TF 1.7 software (AB Sciex, Concord, ON, Canada, 2016), while data processing was carried out using PeakView^®^ software version 2.2 (AB Sciex, Concord, ON, Canada, 2016). The calibration curve of CBDA (y = 2 × 10^8^x + 10^6^) was prepared, and used to quantize the compound in the matrices. Cannabidiolic acid was purified and chemically characterized according to Formato et al. [27]. Briefly, the adopted protocol involved a solid/liquid extraction accelerated by ultrasounds, with chloroform/*n*-hexane (1:1, *v*/*v*) solution as extractant. Then, normal-phase preparative thin-layer chromatography (PTLC) was applied, eluting with the organic phase of a biphasic CHCl_3_/MeOH/H_2_O (13:7:7, *v*/*v*/*v*) solution, followed by reverse-phase (RP-18) TLC, using a MeCN/H_2_O (4:1, *v*/*v*) solution as the mobile phase. CBDA purity was verified by UHPLC-ESI-Q*q*TOF-MS, using the experimental parameters described above. Furthermore, HPLC-UV analysis was performed, by using a HPLC 1260 INFINITY II system, equipped with DAD-UV-visible detector (Agilent, Santa Clara, CA, USA).

The pure compound was stored at −20 °C until cytotoxic and anti-microbial screenings were carried out.

### 3.2. Cell Culture

Human HaCaT epidermal keratinocyte cell line was purchased from ATCC (American Type Culture Collection). Cells were cultured in DMEM high glucose medium supplemented with 10% Fetal Bovine Serum, 50.0 U/mL penicillin, and 100.0 μg/mL streptomycin, at 37 °C in a humidified atmosphere containing 5% CO_2_.

### 3.3. MTT Cell Viability Assay

Cell viability was measured by the 3-(4,5-dimethylthiazol-2-yl)-2,5-diphenyltetrazolium bromide (MTT) assay [12]. HaCat cells were seeded at 1.5 × 10^4^ cells per well in 96-well plates. Twenty-four hours after seeding, cells were treated with CBDA at 0.1, 0.25, 0.5, 1, 2.5, 5, 10, 25, 50 and 100 µM, in the medium for 24 and 48 h exposure times. As control, HaCaT cells were incubated in the medium. After incubation times, the cells were stained with MTT dye solution (0.5 mg/mL) and incubated for 4 h. Subsequently, the MTT-formazan crystals were solubilized in dimethyl sulfoxide (150 μL) and absorbance was read at 570 nm by using a Tecan SpectraFluor (Männedorf, Switzerland) fluorescence and absorbance reader. The cell viability was expressed as a percentage of mitochondrial redox activity (±standard deviation, SD) of CBDA-treated cells in respect to untreated control.

### 3.4. SRB Cell Viability Assay

Cell density determination by sulforhodamine B (SRB) was carried out seeding 1.5 × 10^4^ HaCaT cells per well in 96-well plates [57]. Twenty-four hours after seeding, cells were treated with CBDA at 0.1, 0.25, 0.5, 1, 2.5, 5, 10, 25, 50 and 100 µM (final concentration level in the volume of 100 μL), in the medium for 24 h exposure time (12 wells for each treatment). Untreated HaCaT cells were also incubated (12 wells). At 24 h of incubation, after medium removal, cells were fixed with ice-cold trichloroacetic acid (TCA, 10% (*w*/*v*), 40 µL) for 1 h at 4 °C. Then, plates were washed with distilled water, and dried. Then, 50 µL of sulforhodamine B (SRB, 0.4% (*w*/*v*) in 1% aqueous acetic acid) solution were added to each well, and the plates were incubated at room temperature for 30 min. After the unbound dye removal, by washing the plates with 1% aqueous acetic acid, plates were dried. Thus, the bound SRB was solubilized with unbuffered Tris Base (100 µL; 10 mM, pH 10.5), and the absorbance at 570 nm was measured using a Tecan SpectraFluor fluorescence and absorbance reader. The cell viability inhibition was calculated as a percentage (±standard deviation, SD) of CBDA-treated cells with respect to untreated control.

### 3.5. LDH Cytotoxicity Assay

Lactate dehydrogenase (LDH) activity was measured by using the Cytotoxicity LDH Assay Kit-WST (Dojindo Laboratories, Munich, Germany), according to the instructions of manufacturer. Briefly, HaCat cells were seeded at 1.5 × 10^4^ cells per well in 96 well plates. Twenty-four hours after seeding, cells were treated with CBDA at 0.1, 0.25, 0.5, 1, 2.5, 5, 10, 25, 50 and 100 µM (final concentration level in the volume of 100 μL), in the medium for 24 h exposure time (12 wells for each treatment). Untreated HaCaT cells were also incubated (12 wells). After incubation times, a high control was established by adding 10 μL of the lysis buffer to 6 wells for each treatment and to 6 wells of untreated cells. The plate was incubated at 37 °C for 30 min in the CO_2_ incubator, and then the working solution (100 μL) was added. The mixtures reacted for other 30 min, after that the stop solution (50 μL) was added, and the absorbance was read at 490 nm by using a Tecan SpectraFluor fluorescence and absorbance reader. The LDH cytotoxicity was expressed using the following equation: ((test compound − low control)/(high control − low control)) × 100 [57].

### 3.6. Cytokine Analysis

HaCat cells were seeded at 3.0 × 10^5^ cells per well in 6-well plates. After 24 h, cells were treated with CBDA at 2.5, 5, and 10 µM concentration levels, in free-serum medium, for 48 h exposure time. Following cell harvesting, supernatants were collected and stored at −80 °C until use. Cytokines, chemokines and growth factors levels were evaluated using a Bio-Plex MAGPIX Multiplex Reader system (Bio-Rad, Milan, Italy) equipped with a Bio-Plex Manager software v 6.1 (BioRad) according to manufacturer’s instructions [58]. All washing steps were performed on the Bio-Plex magnetic wash station (BioRad). Measurements were performed in triplicate on samples (50 µL) using Bio-Plex Pro™ Human Cytokine 27-plex Assay (Bio-Rad). Standard curves optimization and the calculation of analyte concentrations were performed by using the Bio-Plex Manager software. Data were expressed as mean ± SD.

### 3.7. Bacterial Strains and Growth Conditions

CBDA was tested against *Escherichia coli* ATCC 13762, *Staphylococcus aureus* ATCC 6538P, *Pseudomonas aeruginosa* ATCC 27853 and *Enterococcus faecalis* ATCC 29212. All bacterial strains were cultured on BD Brain Heart Infusion broth (BHI) (BD, Franklin Lakes, NJ, USA) or on BHI agar (OXOID, Basingstoke, Hampshire, England). The microorganisms were stored at −80 °C in BHI broth containing 10% glycerol (*v*/*v*) (Carlo Erba, Reagents, Milan, Italy) until use and the working cultures were activated in the respective broth at 37 °C with shaking at 225 rpm for 15–18 h. Overnight cultures were diluted to the required cell concentration (≈10^6^ CFU/mL) in BHI broth for growth experiments.

#### 3.7.1. Antibacterial Activity Assay—MIC and MBC Determination

The minimum inhibitory concentration (MIC) and the minimum bactericidal concentration (MBC) of CBDA were determined with a modified version of the broth microdilution assay of the Clinical and Laboratory Standards Institute using a final inoculum concentration of 10^5^ CFU/mL. Briefly, overnight cultures were diluted in fresh broth and allowed to grow until an optical density at 600 nm (OD600nm) of 0.5 had been reached. The working concentration of bacteria was incubated with different dilutions of CBDA ranging from 0.0625 to 64.0 µg/mL for 3–5 h at 37 °C. The concentrations of the molecule tested were 0.0625, 0.125, 0.25, 0.5, 1.0, 2.0, 4.0, 8.0, 16.0, 32.0, 64.0 µg/mL. The MIC represented the concentration of the molecule at which no visible bacterial growth was observed, whereas when 99.9% of the bacterial population is killed at the lowest concentration of an antimicrobial molecule, it is called the MBC.

#### 3.7.2. In Vitro Time-Killing Assay

The kinetics of CBDA antimicrobial activity against all ATCC bacterial strains was assessed at CBDA concentrations corresponding to MIC, 2X MIC, 4X MIC. For time killing studies, the bacterial strains were grown in BHI broth overnight at 37 °C, diluted and sub-cultured until late-log phase containing approximately 10^6^ CFU/mL (adjusted by spectrophotometer to OD600nm) and were centrifuged at 3.000 rpm for 10 min. The pellets were washed and diluted 1:100 in PBS 1X and then incubated at 37 °C with and without CBDA. An aliquot of each sample was taken at baseline and after 1, 2, 3, and 5 h of incubation; subsequently serially diluted in PBS 1X and seeded on BHI agar [59]. The plates were incubated for 24 h at 37 °C and viable bacterial counts were performed by the CFU method [59]. Time-kill curves were plotted graphically as log_10_ CFU/mL vs. time. Each assay was performed in triplicate and all data were expressed as the mean ± SD of three individual experiments.

### 3.8. Biofilm Formation Assay

Biofilm formation assay of *E. coli* ATCC 13762, *S. aureus* ATCC 6538P, *P. aeruginosa* ATCC 27853 and *E. faecalis* ATCC 29212 was conducted in 96-well plates. Bacteria from overnight cultures were diluted and growth until 0.5 McFarland. The bacteria were diluted 1:100 and plated in each well containing 200 µL of the BHI broth. To evaluate the impact of the CBDA on biofilm formation, the medium was supplemented with different concentrations of cannabis extract equal to MIC, 2X MIC, 4X MIC and incubated overnight. At the end of the incubation, the medium was removed; the biofilms were washed twice with PBS and colored with crystal violet (1%) for 30 min, then resuspended in 200 µL of absolute ethanol. Optical density measurements were performed with a spectrophotometer (Enspire, Perkin Elmer, Winter Street, Waltham, MA, USA) at 595 nm. Bacteria incubated with medium alone were used as negative controls, while positive controls were bacteria incubated in medium supplemented with gentamicin 0.2 µg/mL. *S. aureus* and *P. aeruginosa* biofilm formation was assessed also through imaging. After medium was supplemented with different concentrations of CBDA (4.0, 8.0, 16.0 µg/mL for *S. aureus* and 8.0, 16.0, 32.0 µg/mL for *P. aeruginosa*) and incubated overnight, biofilms were stained with a FilmTracer LIVE/DEAD biofilm viability kit (Thermo Fisher Scientific, Third Avenue, Waltham, MA, USA) according to the manufacturer’s instructions. Images were acquired by Cell Discoverer 7, Zeiss. Each assay was performed in triplicate and all data were expressed as the mean ± SD of three individual experiments.

### 3.9. SYTOX Green Uptake Assay Using Fluorescent Spectroscopy

The effect of CBDA on the *S. aureus* ATCC 6538 and *E. coli* ATCC 13762 membrane integrity was assessed by measuring the extent of intracellular accumulation of SYTOX green. Cells from mid-log-phase were collected, washed and resuspended in 10 mM phosphate buffer. The final density was adjusted to 5 × 10^7^ CFU/mL. Cells were then treated with CBDA at concentrations equal to MIC, 2X MIC, 4X MIC (4.0, 8.0, 16.0 µg/mL for *S. aureus* and 0.5, 1.0, 2.0 µg/mL for *E. coli*) in presence of 200 nM SYTOX green (Invitrogen, Waltham, MA, USA). Enhancement in SYTOX green fluorescence, a direct measure of the extent of membrane permeabilization, was monitored in a fluorescence spectrophotometer (Enspire, Perkin Elmer). Excitation and emission wavelengths used were 503 and 523 nm, respectively.

## 4. Conclusions

In spite of a not so glorious past, Cannabidiolic acid is a bioactive compound worthy of attention in the industrial hemp nutraceutical and cosmeceutical scenario. Edible hemp oil benefits from the presence of this compound, which, together with other nutritionally valuable compounds, makes this oil a precious functional product. On the other hand, the possibility of recovering the compound from hemp food chain waste opens up new insights into the exploitation of its anti-inflammatory and antimicrobial power for skin health.

## Figures and Tables

**Table 1:** Antimicrobial activities of CBDA against ATCC strains. ^a^ MIC, minimum inhibitory concentration values are expressed as concentration of CBDA. ^b^ MBC, minimum bactericidal concentration values are expressed as concentration of CBDA.

	MIC ^a^ (µg/mL)	MBC ^b^ (µg/mL)
*E. coli* ATCC 13762	0.5	8
*S. aureus* ATCC 6538	4	16
*P. aeruginosa* ATCC 27853	8	32
*E. faecalis* ATCC 29212	4	32

## Data Availability

The data are included in this manuscript and in its Supplementary Materials.

## Supplementary Materials

The following supporting information can be downloaded at: https://www.mdpi.com/article/10.3390/molecules27082566/s1, Figure S1. TOF-MS and MS/MS spectra of CBDA detected in leaf hemp sample and cold-pressed hemp seed oil. Red arrows highlight the corresponding peaks in the TICs (Total Ion Chromatograms). Figure S2. UHPLC-TOF-MS chromatogram of purified CBDA, acquired using the same experimental conditions of hemp leaf extract. Figure S3. (A) HPLC-UV chromatogram recorded at 266 ± 4 nm; (B) HPLC-UV chromatogram recorded at 306 ± 4 nm; (C) UV spectrum of purified CBDA.

## Sample Availability

Samples of the compound are available from the authors.
