# Supplementary material for: Cannabidiolic acid in Hemp Seed Oil Table Spoon and Beyond
Source: Molecules. 2022 Apr 15;27(8):2566. doi: 10.3390/molecules27082566 (PMC9029873; doi:10.3390/molecules27082566)
Supplement: Supplementary file 1 [file molecules-27-02566-s001.zip › molecules-1654016-supplementary.pdf]

Supplementary materials

Cannabidiolic acid in hemp seed oil table spoon and beyond

Ersilia Nigro[1,2,†], Maria Tommasina Pecoraro[1,†], Marialuisa Formato[1], Sara Ragucci[1], Simona Piccolella[1], Marta Mallardo[1,2], Rosita Russo[1], Antimo Di Maro[1], Aurora Daniele[2,3,*] and Severina Pacifico[1,*]

[1] Dipartimento di Scienze e Tecnologie Ambientali, Biologiche, Farmaceutiche, Università della Campania "Luigi Vanvitelli", 81100, Caserta, Italy.
[2] CEINGE, Biotecnologie Avanzate Scarl, Napoli.
[3] Dipartimento di Medicina Molecolare e Biotecnologie Mediche, "Federico II" Università degli Studi di Napoli, Napoli, 80131, Italy.
* Correspondence: severina.pacifico@unicampania.it; aurora.daniele@unina.it
† These authors equally contribute to the manuscript

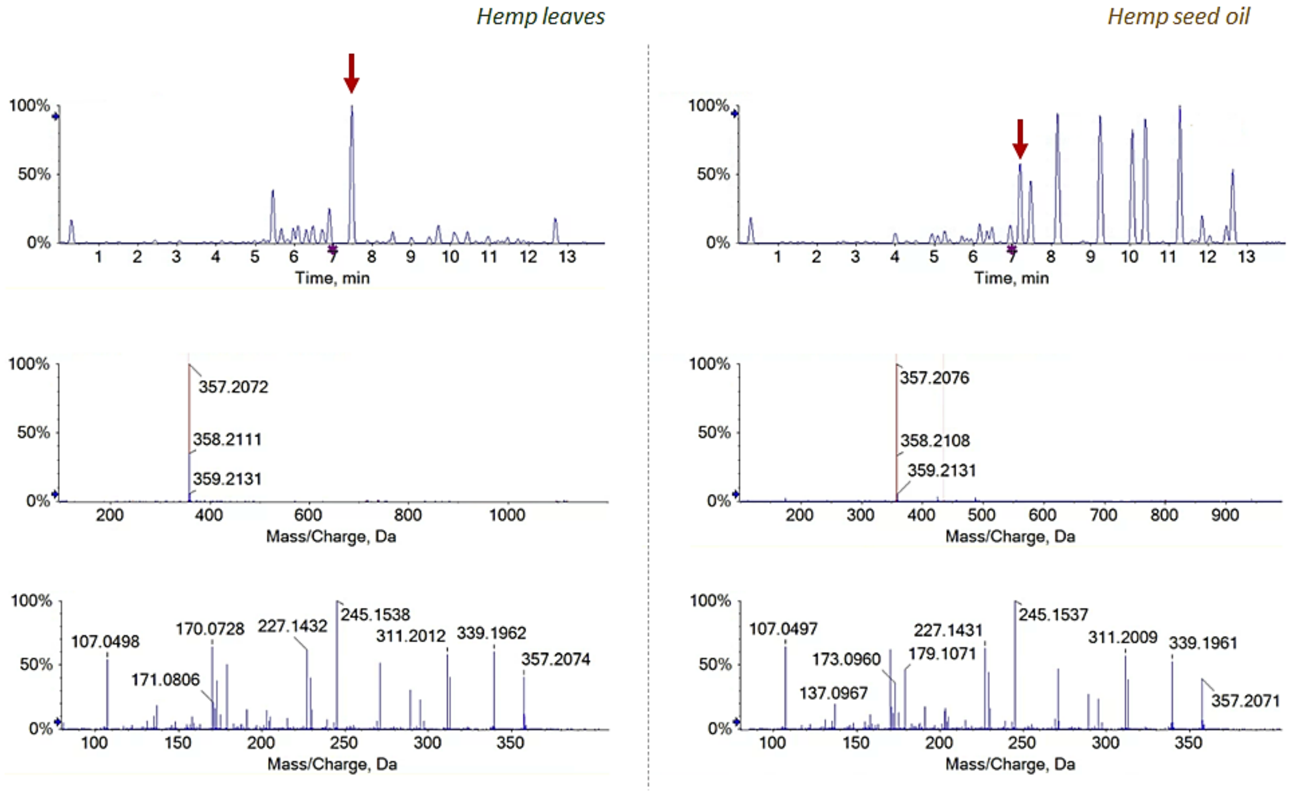

Figure S1. TOF-MS and MS/MS spectra of CBDA detected in leaf hemp sample and cold-pressed hemp seed oil. Red arrows highlight the corresponding peaks in the TICs (Total Ion Chromatograms).

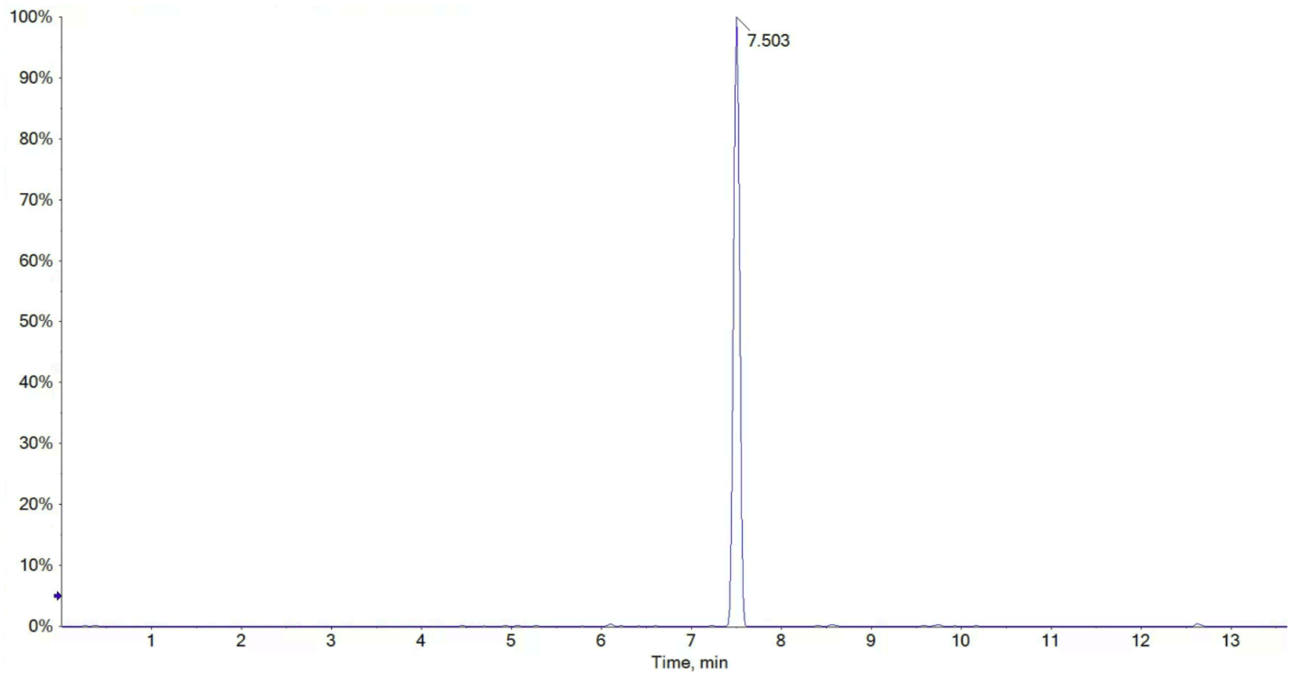

Figure S2. UHPLC-TOF-MS chromatogram of purified CBDA, acquired using the same experimental conditions of hemp leaf extract.

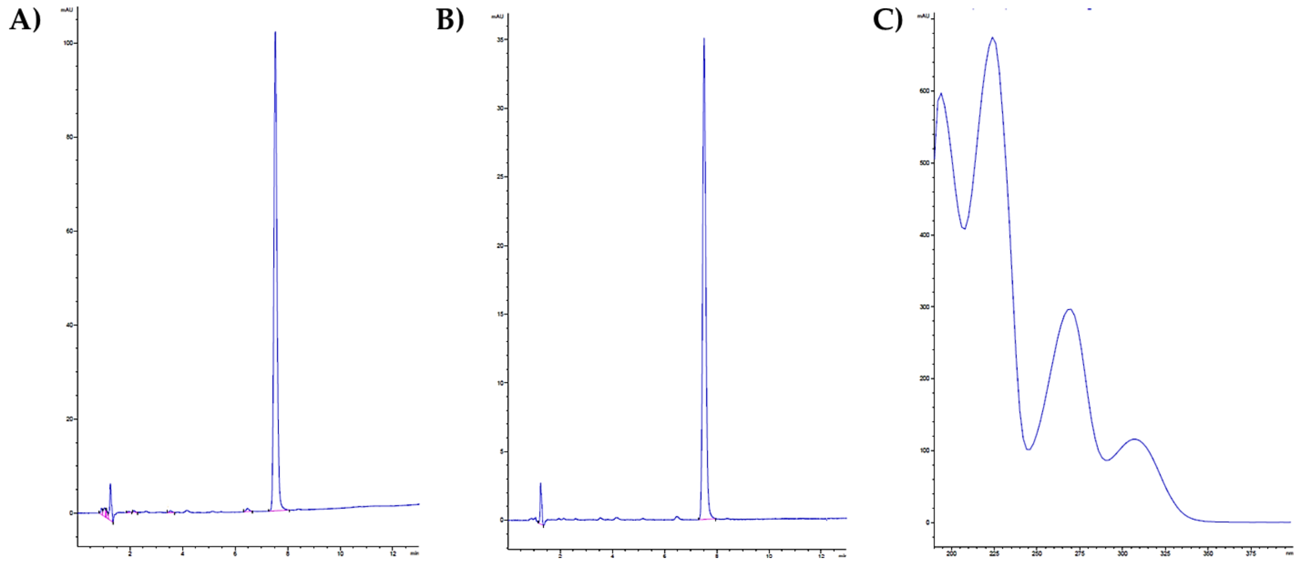

Figure S3. A) HPLC-UV chromatogram recorded at 266±4 nm; B) HPLC-UV chromatogram recorded at 306±4 nm; C) UV spectrum of purified CBDA.
